# Clemastine promotes recovery of neural function and suppresses neuronal apoptosis by restoring balance of pro-inflammatory mediators in an experimental model of intracerebral hemorrhage

**DOI:** 10.7150/ijms.51150

**Published:** 2021-01-01

**Authors:** Cheng Zhi, Shulian Zeng, Yuan Chen, Degui Liao, Miaoling Lai, Zhaotao Wang, Yezhong Wang, Shiyin Xiao

**Affiliations:** 1 Department of Pathology, the Second Affiliated Hospital of Guangzhou Medical University, Guangzhou 510260, China.; 2 Department of Neurology, Heyuan People's Hospital, Heyuan, 517000 China.; 3 Department of Respiratory and Critical Care Medicine, Nanfang Hospital, Southern Medical University, Guangzhou 510515, China.; 4 Institute of Neuroscience, the Second Affiliated Hospital of Guangzhou Medical University, Guangzhou 510260 China.

**Keywords:** Intracerebral hemorrhage, Clemastine, histamine receptor H1

## Abstract

Intracerebral hemorrhage (ICH) represents a common acute cerebrovascular event that imparts high rates of disability. The microglia-mediated inflammatory response is a critical factor in determining cerebral damage post-ICH. Clemastine (CLM) is a histamine receptor H1 (HRH1) antagonist that has been shown to modulate the inflammatory response. However, the effects of CLM on ICH and the underlying mechanism remain to be determined. This investigation reveals that CLM resulted in reduction of cerebral hematoma volume, decreased cerebral edema and lower rates of neuronal apoptosis as well as improved behavioral scores in an acute ICH murine model. CLM treatment was noted to decrease pro-inflammatory effectors and increased anti-inflammatory effectors post-ICH. In addition, CLM reduced the deleterious effects of activated microglia on neurons in a transwell co-culture system. Our findings show that CLM likely mediates its therapeutic effect through inhibition of microglia-induced inflammatory response and apoptosis, thereby enhancing restoration of neuronal function.

## Introduction

Intracerebral hemorrhage (ICH) is the result of a sudden rupture of cerebral blood vessels and carries with it high morbidity and mortality rates. 20-30% of stroke cases in Asia were reported to involve ICH, while this number lies at approximately 10-15% in countries such as the United States, Europe and Australia 1, 2. The advent of several neuroprotective strategies has failed to result in higher survival rates or improved quality of life post-ICH 3, 4. Therefore, further studies of the pathogenesis of ICH and the discovery of safe and effective therapeutic regimens are of utmost importance for patients with this debilitating condition.

The pathogenesis of ICH involves both primary and secondary injury. The surrounding brain tissue is compromised via direct compression of the enlarging hematoma, resulting in primary injury. This phenomenon is augmented by the formation of cellular edema, which develops as a result of inflammatory processes triggered by the presence of extravasated blood 2. Secondary injury is mediated by neuroinflammation caused by activation of resident microglia, leading to free radical formation and neuronal apoptosis 5-7. The presence of extravasated blood is a critical trigger of the microglial-activated cerebral inflammatory response 8. A robust activation of microglia may lead to infiltration of a large amount inflammatory cytokines such as free radicals, chemokines, tumor necrosis factor-α (TNF-α), and interleukin-1β (IL-1β) - all of which impart significant neurotoxicity. This line of evidence leads to the conclusion that attenuating microglial activation may serve as a suitable therapeutic strategy in the management of ICH.

Clemastine (CLM) is a first-generation histamine HRH1 antagonist that demonstrates potent anti-inflammatory effects as well as enhances oligodendrocyte differentiation both *in vitro* and *in vivo* 9. A recent study confirmed that CLM restores the imbalance of inflammatory markers in mice hippocampi with depressive disorder 10. However, the effects of CLM on ICH reminds unknown and the underlying mechanism needs to be explored.

We designed this study to determine whether CLM could improve behavioral recovery after ICH and to determine its how it regulates the acute inflammatory response in ICH.

## Materials and Methods

### Animals and Experimental Design

The investigations were carried out in compliance to standards predetermined by the Animal Ethics Committee of the Guangzhou Medical University. 8 to 10 weeks old male C57BL/6 mice that weighed between 20 to 25 g were procured from the Animal Center of South Medical University. The animals were reared with a 12 hours light/dark cycle at 23-25 °C with unlimited water and food. The mice were randomized to three cohorts: sham (needle insertion only), ICH + vehicle (ICH+DMSO) and ICH + CLM (ICH + CLM treatment) groups.

### Chemicals, reagents and antibodies

CLM (Sigma-Aldrich, St. Louis, MO, USA) was diluted in DMSO and kept at 4 °C. Antibodies against iNOS, Agr-1, TNFα, IL-1β and GAPDH were purchased from Cell Signaling Technology (Beverly, MA). Fetal bovine serum (FBS) and Dulbecco's modified Eagle medium (DMEM) were manufactured by Gibco (Grand Island, USA).

### Establishment of an ICH model and treatment protocol

Mice were anaesthetized with 1% pentobarbital sodium (50 mg/kg) intraperitoneally. After being anaesthetized, mice were secured onto a stereotaxic instrument. Skull burr holes based on the stereotaxic coordinates of the Paxinos and Franklin mouse brain atlas (0.2 mm posterior, 2.8 mm ventral, and 2.2 mm lateral to the bregma) were drilled prior to insertion of a needle into the striatum. Bacterial collagenase VII (0.1 U in 0.4 μl; Sigma) was infused at a rate of 400 nl/min over 1 minute into the right striatum. After 10 minutes, the needles were gently removed. 15 minutes after this, either CLM diluted in 200 μl normal saline, or equal vehicle volume were given by intraperitoneal injection to the mice. All experiments were performed in threes.

### Modified Neurological Score Test

The Modified Garcia Score was used to assess the degree of neurological dysfunction in mice 11. Mice were first trained at three days before the stereotaxic procedure. Two independent observers blind to the mice cohorts evaluated the neurological status of the mice at 24 h, 48 h and 72 h of ICH. Neurological deficit was ranked on a scale of 0-18, with normal at 0 and 18 indicating impaired function.

### Determination of Lesion Volume

All mice were decapitated under deep anesthesia three days after induction of ICH. Brains were harvested in order to produce 1-mm thick coronal sections. Aerial digital images of adjacent sections from each brain were attained. Volumes of the lesions were derived using the Image Pro-Plus software (Media Cybernetics) by multiplying the area of blood clots on each slice by the distance between sections.

### Cerebral edema assessment

Cerebral edema was also measured at three days post-ICH induction in order to capture the time of maximal edema. The (wet-dry)/(wet brain weight) formula to determine brain water content was used 12.

### Reverse transcription-quantitative PCR (RT-qPCR)

Real-time PCR was used to determine mRNA levels of iNOS, Arg-1, TNF-α and IL-1β. Brain tissue surrounding the haemorrhage (diameter of 3mm) was extracted and treated with TRIzol reagent (Life technologies, CA, USA) prior to precipation using chloroform/isopropanol 13. PrimeScript RT Reagent Kit (TaKaRa Biotechnology Co. Ltd., Dalian, China) mixed with the UltraSYBR master mix (Mixture-with ROX; CWBIO, Beijing, China) were utilized to produce cDNA. The following primer sequences are shown below in Table 1.

### Western blotting

Total protein was extracted from isolated peri-hemorrhage brain tissue by homogenization with lysis buffer (CWBIO, China) and protease inhibitor. The tubes were kept for 30 minutes on ice before being subjected to 15 minutes of centrifugation at 14000×g at 4 °C. The Micro BCA Protein Assay (Thermo Fisher Scientific Inc., MA, USA) was used to quantify protein concentrations. The extracted proteins were then electrophoresed with 10% SDS-PAGE gel and blotted onto Poly Vinylidene Difluoride (PVDF) membranes (Millipore, MA, USA). These membranes were immersed in 5% bovine serum albumin (BSA; Sigma, St. Louis, MO, USA) for 1 h to block endogenous reactions and incubated overnight with primary at 4 °C. Secondary antibodies were then added the next morning at room temperature for 1 h. The membranes were probed with specific primary antibodies and HRP-conjugated secondary antibodies. The blots were visualized with ECL luminescence reagents.

### Immunofluorescence

Fixed brain tissues were sliced into 20 μm-thick coronal sections using a freezing sledge microtome. Standard techniques were used for immunofluorescence staining of cryostat sections and for fluorescence microscopy. The samples were washed thrice with 1× PBS and immersed for an hour in normal goat serum containing 0.3% Triton X-100. The appropriate primary antibodies diluted in 0.01 M PBS were then added into the samples and left overnight at 4 °C. The following morning, the samples were again rinsed thrice with PBS before being left to incubate at room temperature with Alexa Fluor 555-conjugated secondary antibody (Invitrogen, Carlsbad, CA, USA) for 45 min. The sections were washed three times with 1×PBS before being counter stained by DAPI (Sigma-Aldrich, St. Louis, MO, USA, 1:500) for 10 min in order to visualize the nuclei. Imaging was obtained after samples were again washed and air-dried. A light or fluorescence microscopy under a confocal microscope (LSM 510, META Laser Scanning Microscope, Zeiss) was used to capture images.

### *In situ* cell death detection using TUNEL staining

*In situ* DNA fragmentation was assessed to quantify the rate of apoptotic cell death. An *In situ* Cell Death Detection kit (Roche, Germany) that was able to carry out TUNEL staining was utilized based on protocols stipulated by the manufacturer. Brain tissues were fixed and sliced into 20 μm-thick coronal sections with a sledge microtome. Sections were incubated for 2 minutes with permeabilization solution comprising of 0.1% Triton X-100 and 0.1% sodium citrate at 4 °C. Sections were first rinsed and dried. TdT enzyme reaction buffer that was labeled dUTP was then incubated at 37 °C for 1 hour with the rinsed and dried sections. Stained cells were then observed under a microscope.

### Primary neuron culture

Primary cortical neurons were extracted from 14-days-old mouse fetuses. Blood vessels and meningeal tissue were removed from the brains before they were immersed in ice-cold HBSS buffer with 20% FBS. Samples were exposed to accutase that had 100U/ml DNAse I (Gibco, USA) for 15 minutes in order to digest and separate the cells. Cells were centrifuged and resuspended in neurobasal medium enhanced with 1% glutamine and 2% B27 before being seeded onto flasks pre-coated with 100 μg/ml poly D-lysine. The neurons were placed in an incubator at 37 °C under 5% CO_2_ for 3 days. Nonneuronal cell proliferation was blocked with Ara-C. The cells were harvested after being cultured for 14 days.

### Transwell co-culture

The microglial cells were co-cultured with primary cortical neurons using transwell inserts. 2×10^4^ BV2 cells and 1×10^4^ neurons were respectively seeded in the upper and lower chamber of the transwell in neurobasal medium supplemented with 1% glutamine and 2% B27. The cells were cultured under 5% CO_2_ at 37 °C.

### Statistical analyses

The SPSS 20.0 was used to perform statistical analysis. Normal test and homogeneity test of variance were checked for in data acquired from each group. All data are shown as the mean ± SD. One-way analysis of variance (ANOVA) allowed for multiple group comparison followed by LSD or Dunnett's post hoc test. A probability value<0.05 was taken to indicate significance.

## Results

### CLM restored neurological deficits, shrank lesion volumes and alleviated cerebral edema following ICH induction

**Figure 1:**
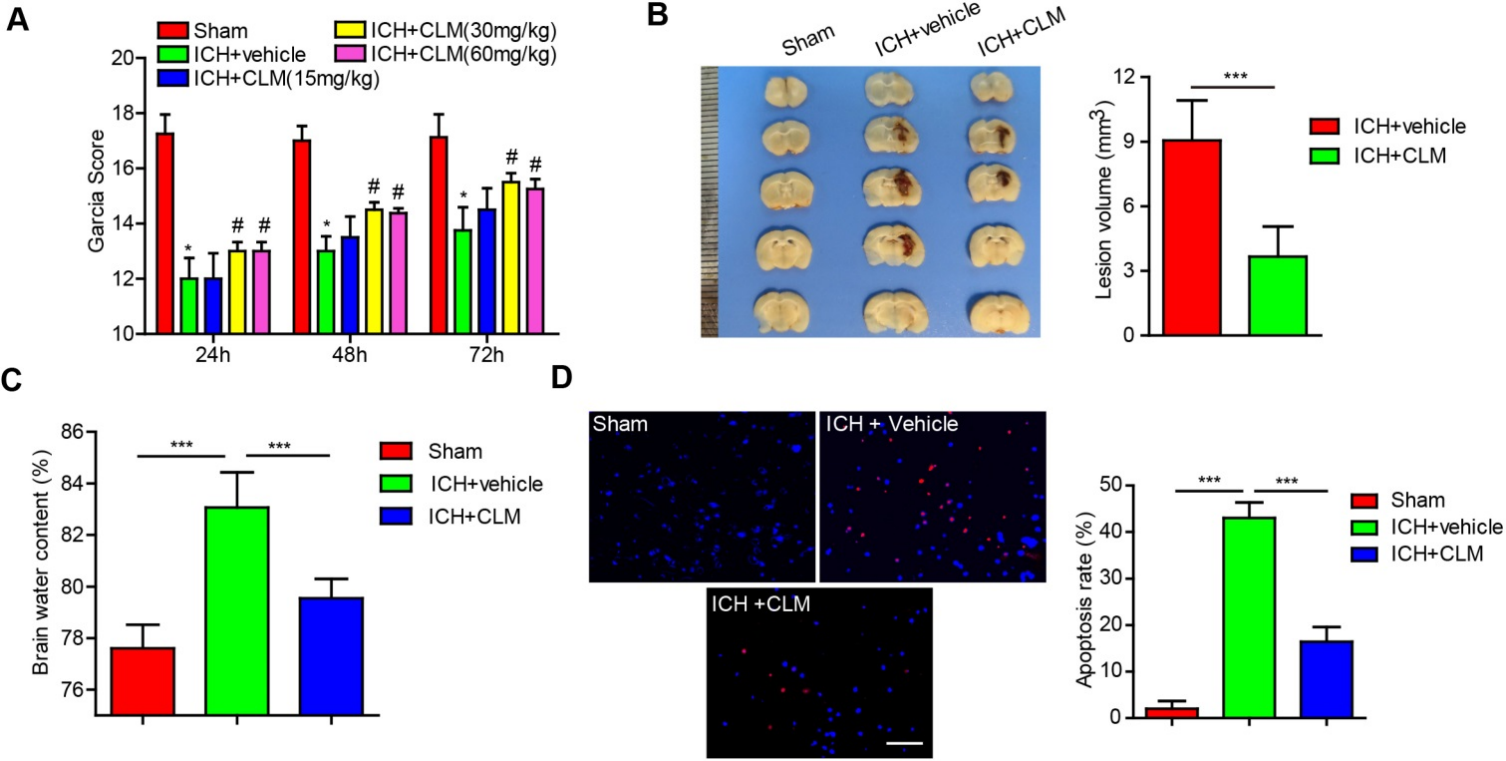
** Intraperitoneal injection of CLM restored or accelerated neurological function. (A)** Garcia test scores of mice cohorts at 24, 48 and 72 hours post-ICH or sham-operation. **(B)** Coronal sections show lesion areas of mice brains belonging to three different groups (Left), and quantitative analyses of the lesion volumes 72h (n = 5, each group) post-ICH(Right). **(C)** Cerebral water content of the ipsilateral hemorrhagic hemispheres was assessed at 72h (n = 5, each group) post-ICH in each group. **(D)** Percentage of apoptotic cells after 72h (Bar = 50 µm). *p<0.05, **p<0.01, ***p<0.001, in contrast to the sham group; #*p*<0.05, #in contrast to the ICH + vehicle group.

To explore the effect of CLM after intracerebral hemorrhage in mice, the degree of neurological deficits was first assessed. We treated the ICH mice models with different doses of CLM and evaluated their neurological function. There did not appear to be any significant differences observed in the ICH + Vehicle group when the CLM dose was less than 30 mg/kg. Additionally, when the VB dose was more than 30 mg/kg (for example, 60 mg/kg), there did not appear to be any significant improvements in neurological function compared to the 30 mg/kg group (Fig. 1A). We found 30 mg/kg CLM to be the optimal dose to achieve maximal protective effects. Thus, we chose to use 30 mg/kg CLM to conduct the following experiments.

Next, the effects of CLM on the volume of cerebral lesion and the degree of cerebral edema were assessed at 3 days post-ICH. The lesion volumes, as well as brain water content, were significantly reduced in ICH +CLM group in contrast to the ICH group (Fig. 1B-C).

### CLM inhibited cell apoptosis in mice with ICH

To confirm the impact of CLM on apoptosis after ICH, the levels of apoptosis were detected by TUNEL staining. The results indicated that CLM markedly lowered the proportion of apoptotic cells in the ICH +CLM group in the periphery after cerebral hemorrhage (Fig. 1D) compared to the ICH group.

### CLM decreased the levels of inflammatory effectors in brains of ICH mice models

**Figure 2:**
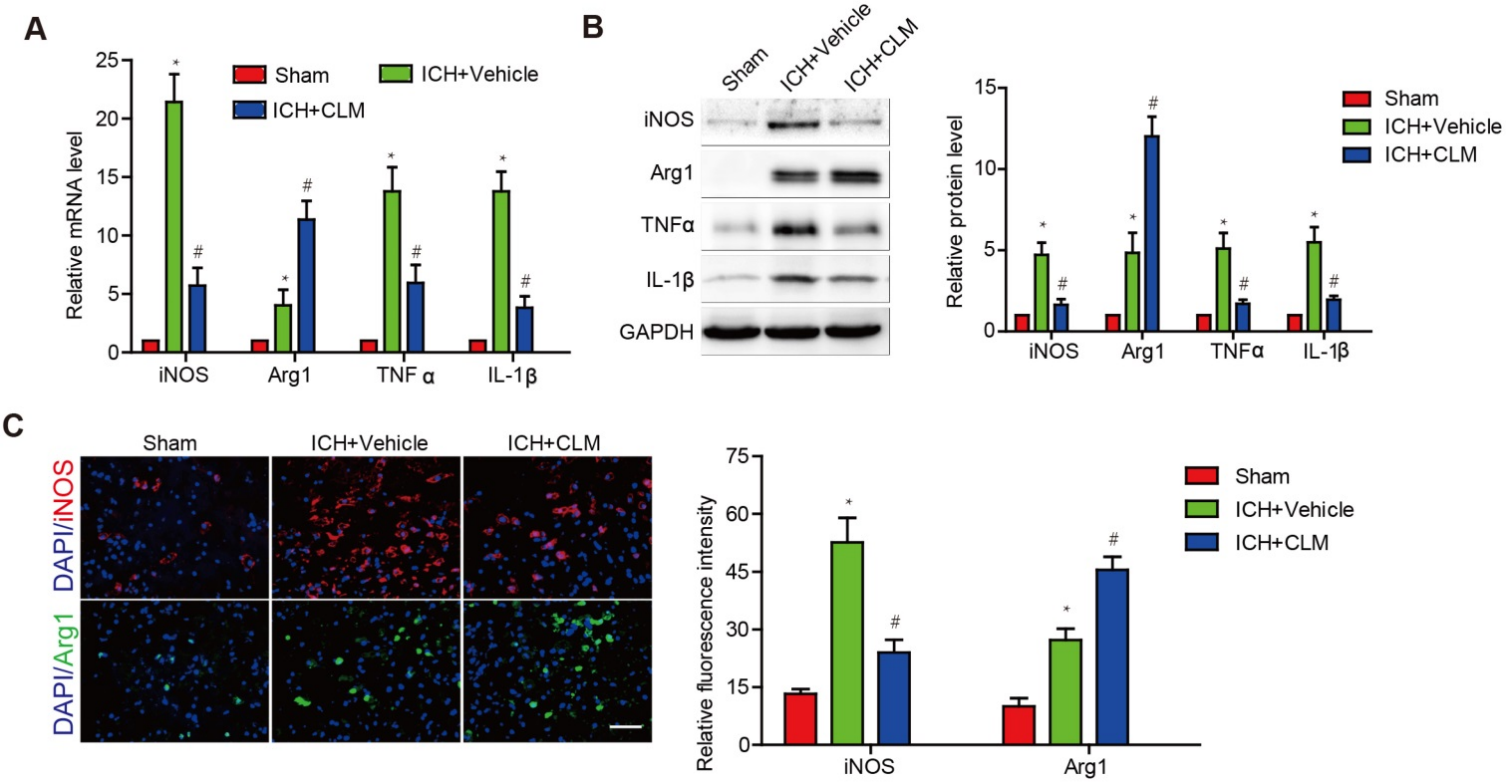
** The anti-inflammatory effect of CLM in peri-hemorrhagic areas.** Inflammatory effectors iNOS, Agr-1, TNF-α and IL-1β were detected at gene and protein levels at 72h by RT-PCR **(A)** and western blotting **(B)**. **(C)** Quantification of iNOS and Agr-1 protein expressions in the lesions after 72 h (scale bar = 50 µm). *p<0.05, in contrast to the sham group; #*p*<0.05, in contrast to the ICH + vehicle group.

To evaluate the inflammatory response in the brains of ICH mice following CLM administration, we analyzed the levels of inflammatory effectors in brain tissue at 3 days post-ICH. Pro-inflammatory effectors iNOS, TNF-α and IL-1β were at high levels in contrast to those of the sham group. The ICH+CLM group showed detectable down-regulation in iNOS, TNF-α and IL-1β mRNA and protein levels in contrast to group that was not given CLM. There was higher expression of anti-inflammatory effectors Arg1 in the ICH + CLM group than in the CLM group (Fig. 2A-B). In addition, similar results were also observed by immunofluorescence analysis of iNOS and Agr-1 expression (Fig. 2C). These results indicated that CLM inhibited the inflammatory response of cerebral tissue in ICH mice models.

### CLM attenuated the levels of inflammatory effectors in RBC-lysis induced ICH

**Figure 3:**
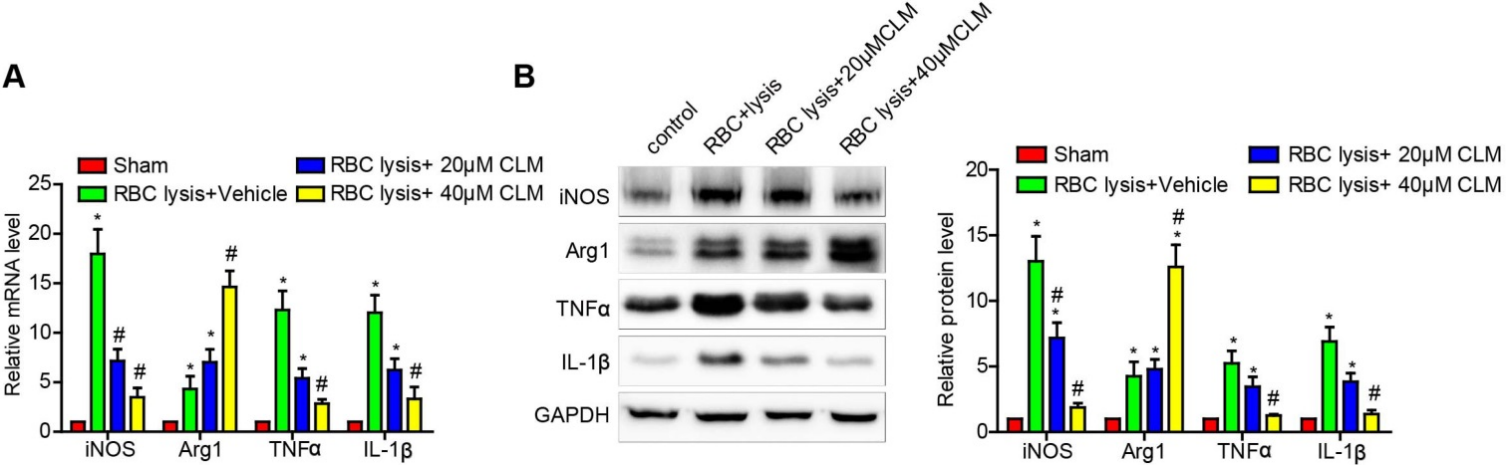
** CLM reduced RBC lysis-induced microglial activation *in vitro*.** iNOS, Arg-1, TNF-α and IL-1β were detected at gene and protein levels at 72h by RT-PCR **(A)** and Western blotting. **(B)** Microglial cells co-cultured with primary neurons in the presence of lysed RBCs and treated with CLM. *p<0.05, in contrast to the sham group; #*p*<0.05, in contrast to the ICH + vehicle group.

Microglial cells have proven to be critical effectors of the post-ICH inflammatory response. The above results showed that CLM decreased ICH-induced inflammation *in vivo*. To further test our findings, we stimulated microglial cell BV2 with lysed murine RBC and further observed their responses in the presence and absence of CLM treatment. The results showed that CLM significantly decreased levels of pro-inflammatory effectors iNOS, TNF-α and IL-1β, while increasing levels of the anti-inflammatory effectors Arg1 both at the mRNA and protein level in a dose-dependent manner (Fig. 3). These findings suggested that CLM suppressed microglia activation *in vitro*.

**Figure 4:**
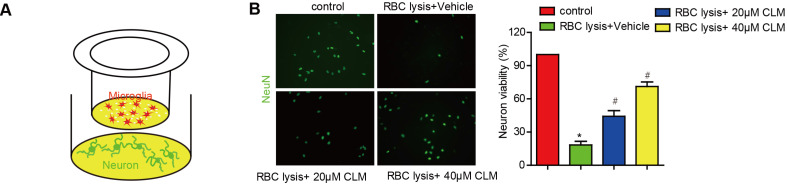
** CLM reduced neuronal damage induced by RBC lysis *in vitro*. (A)** A schematic diagram of microglial cells co-cultured with primary neurons in the presence of lysed RBCs using transwell inserts. **(B)** Images of NeuN+ (green) primary neurons co-cultured and treated as described (scale bar = 50 µm) and their viability **(C).** *p<0.05, in contrast to the sham group; #*p*<0.05, in contrast to the ICH + vehicle group.

We then scrutinized the impact of CLM on microglia-mediated neuronal toxicity. Post-ICH neurons were co-cultured with microglia cells, and stimulated with lysed RBC cells with or without CLM treatment (Fig. 4A). The presence of lysed RBC reduced neuronal viability if CLM treatment was administered. CLM was able to preserve neuron populations in a dose-dependent manner (Fig. 4B). These results indicated that CLM protected against neuronal apoptosis induced by microglia cell activation.

## Discussion

Intracerebral hemorrhage is an epidemiologically significant condition that is severely detrimental to human health and life 14. Existing clinical trials are mostly aimed at investigating ICH-mediated primary cerebral injury, however, none have demonstrated an approach which substantially improves clinical outcomes 15-17. Evidence suggests that the subsequent inflammatory cascade is a critical contributor of post-ICH complications 18, 19. Hence, it is important to investigate effective therapeutic strategies that target the secondary inflammatory response in ICH.

Cerebral edema carries a significant role in secondary injury post-ICH 20. It leads to increased intracranial pressure and eventually cerebral herniation, if severe. Methods to reduce cerebral edema in a fast and safe manner are urgently needed. Our results first showed that CLM could relieve brain edema, and that CLM treatment post-ICH markedly shrank volumes of the primary lesion. In addition to this, our data showed that CLM could restore neurological deficits and reduce neuronal apoptosis. These results provide a theoretical basis for the usage of CLM for ICH.

An increase in parenchymal hemolytic products produces a pronounced inflammatory response in the neighboring cerebral tissue post-ICH, characterized by inflammatory cell activation and mobilization leading to infiltration of various circulating immune cells and inflammatory cytokine secretion, including tumor necrosis factor-α and interleukin-1β 21. Increased inflammatory cytokines further enhance infiltration of lymphocytes leading to a vicious, self-sustaining inflammatory cycle. Therefore, attenuation of the inflammatory response is critical in promoting neurological recovery. In previous studies, Yuan X et al. reported that CLM protected cardiomyocytes through inhibition of the TLR4/PI3K/Akt signaling pathway 22. Wen-Jun Su, et al. showed that CLM regulated the balance between inflammatory and anti-inflammatory markers within the hippocampus and in the serum of mice with depression 10. In this study, we reported that CLM can significantly inhibit the inflammatory response through downregulation of TNF-α, IL-1β, iNOS and upregulation of the anti-inflammation effector Agr-1 post-ICH.

The release of inflammatory cytokines comes mainly from microglial cells and some researchers have reported that the numbers of cells reached the peak value at 3 days post injury in ICH animal models 23, 24. Microglia can be triggered to release pro-inflammatory mediators that are toxic to neurons 25. Thus, multiple studies have demonstrated the importance of early attenuation of microglial activation. This study demonstrated that CLM suppressed microglial activation and neuroinflammation in a murine ICH model and a co-culture system, indicating that CLM protected the mice from further secondary damage mediated by ICH likely through modulation of the inflammatory response.

CLM, as a histamine HRH1 antagonist, is commonly used to treat the allergic diseases. However, it has been reported that CLM could cure depressive disorders in mice 9. Moreover, it has been demonstrated that CLM could reverse myelination defects and promote functional recovery in hypoxic brain injury in humans 10. In our study, we found that CLM resulted in reduced cerebral inflammatory response in ICH mice models, highlighting that CLM may have a wider range of therapeutic potential outside of treating allergies.

In conclusion, our findings showed that CLM treatment was able to improve overall neurological function in ICH-induced brain damage. CLM is a potent anti-inflammatory and anti-apoptotic agent. Further investigations exploring its potential in the clinical management of ICH and its associated cerebral damage are necessary.

## Figures and Tables

**Table 1:** Primer sequences

Primer	Sequence
β-actin	Forward: 5'-AACCCTAAGGCCAACCGTGAAAAG-3'
	Reverse: 5'-TCATGAGGTAGTCTGTCAGGT-3'
Agr-1	Forward: 5' - CATTGGCTTGCGAGACGTAGAC-3'
	Reverse: 5'- GCTGAAGGTCTCTTCCATCACC -3'
iNOS	Forward: 5'-GAGACAGGGAAGTCTGAAGCAC -3'
	Reverse: 5'- CCAGCAGTAGTTGCTCCTCTTC -3'
IL-1β	Forward: 5'- GGAACCCGTGTCTTCCTAAAG-3'
	Reverse: 5'- CTGACTTGGCAGAGGACAAAG-3'
TNF-α	Forward: 5'-CCAACAAGGAGGAGAAGTTCC-3'
	Reverse: 5'- CTCTGCTTGGTGGTTTGCTAC-3'
